# Multiscale Prediction for Mechanical and Thermal Properties of Needled Composites Considering Pore and Their Application

**DOI:** 10.3390/ma18214855

**Published:** 2025-10-23

**Authors:** Shiyong Sun, Junlong Wang, Hailin Li, Rui Yang, Liming Zhou

**Affiliations:** 1 School of Mechanical Engineering, Dalian University of Technology, Dalian 116024, China; 2 State Key Laboratory of High-performance Precision Manufacturing, Dalian University of Technology, Dalian 116024, China

**Keywords:** manufacturing defects, elastic properties, thermal properties, multiscale method, needled ceramic matrix composites

## Abstract

Needled ceramic composites have great application prospects for high-temperature structural components. However, due to the manufacturing defects, the properties of the composite show significant dispersion, which poses great challenges for predicting the service life. Firstly, X-ray computed tomography was used to determine the pores in the composites, and multiscale models considering the pores were established. Combined with the multiscale method, the elastic modulus was predicted, and the relationship between porosity and elastic modulus was established. Secondly, the thermal diffusion coefficient was predicted. The relationship between porosity and thermal diffusion coefficient was determined. The accuracy of the multiscale method was verified by comparative analysis with the tensile experiment and the thermal diffusion experiment, respectively. Finally, based on the results of the model analysis, the correlation equations between thermal diffusion coefficient, porosity and elastic modulus were established. Thereby, new ideas are provided for the assessment of porosity and elastic properties of the composites.

## 1. Introduction

Needled ceramic matrix composites (N-CMCs) are considered as one of the most promising materials in the aerospace field due to their excellent specific strength, interlayer bonding properties and impressive high-temperature stability [1]. However, the extreme service environment, such as high temperature, high stress and oxidation ablation, places higher requirements for the reliability and safety of composites [2]. Due to the complexity of the manufacturing process, there are a large number of obvious manufacturing defects in N-CMCs, including pores, microcracks and fiber deflection [3]. The structural discontinuity and anisotropy of N-CMCs make their mechanical response complex and difficult to predict, which negatively affects their reliability and durability in engineering applications.

The manufacturing process of N-CMCs is mainly divided into the needling process and the matrix deposition [4]. When the needled bundle is formed, part of the fiber bundle is deflected, and pores of different sizes are formed inside the preform. In the process of matrix deposition, the gaseous precursor diffuses through the pores in the fiber preform, and the premature sealing of the pores on the fiber surface prevents the gas from transferring to the interior, resulting in a density gradient inside the composites [5]. Pores are particularly common in components with a large size, thick walls or complex shapes, which leads to uneven distribution of the matrix and pores between or within fiber bundles and significantly affects the mechanical properties of the materials [6]. It turns out that these defects are difficult to detect through visual inspection of the surface appearance and are often overlooked, posing a threat to the reliability and safety of N-CMC components [7].

X-ray computed tomography (XCT) has great advantages for analyzing the internal microstructure of CMCs. The research on mechanical properties of manufacturing defects using testing, theory and simulation is one of the current research priorities, based on XCT technology. Mei et al. [8] prepared 3D-needled C/SiC composite specimens with density defects and discussed the tensile and compressive properties of the specimens with density defects from the perspective of XCT, IRT and mechanical testing. Y. Gowayed et al. [9] combined XCT to classify the manufacturing defects of CMCs and analyzed their influence on the elastic properties using theoretical prediction methods.

In order to improve the calculation accuracy, the finite element method (FEM), which can realize the fine modeling of defects, has attracted more and more attention. Lin et al. [10] established a FEM model based on CT technology and directly modeled the internal structure of N-CMCs based on micro-CT images, which could reproduce the precise geometric structure of the composites with high precision. Ge et al. [11] measured internal pores using XCT and constructed some complex models with defects to investigate the influence of defects on strength. Li et al. [12] used SEM and XCT to establish a complex model that comprehensively considered pores and combined them with the acoustic emission (AE) technique to evaluate the effect of lay-up mode and porosity. However, such methods also had problems with computational effort. Therefore, to achieve better calculation results, many researchers have investigated various multiscale methods. Wang et al. [13] used XCT technology, homogenization theory and representative volume element (RVE) to develop an analysis method that considered multiscale manufacturing defects and investigated the influence of defects on the stiffness of the composites. Han and Wang et al. [14,15] proposed mesoscale models that used circular arc bundle elements to characterize the deflection of needle and fiber bundles. Although some research achievements have been obtained based on XCT technology, challenges such as large computational effort in observing and modeling large-sized components remain in rapid defect evaluation and property analysis.

Commonly used non-destructive testing (NDT) methods that can achieve rapid detection for CMCs include infrared thermal imaging (IRT) [16,17], terahertz (THz) [18] and ultrasonic testing (UT) [19]. Zhang et al. [18] accurately identified the bonding defects of CMCs using the THz NDT method. G. Meyer et al. [16] successfully applied IRT to non-destructively evaluate the porosity of carbon fiber-reinforced polymer, and the accuracy of IRT in porosity evaluation was verified by comparative analysis with UT. Gao et al. [20] investigated the feasibility of electrical resistance tomography for detecting CMC defects. J.P. Goulmy et al. [21] evaluated the microstructure of CMCs after oxidation using XCT, scanning electron microscopy (SEM), optical microscopy (OM) and other characterization techniques. Liang et al. [7] adopted two nondestructive testing techniques, IRT and XCT, to identify density defects from qualitative and quantitative perspectives, respectively. As mentioned above, the current research, combined with rapid detection methods, mainly focuses on identifying the composite defects. In practical engineering applications, it is more desirable to directly evaluate the properties of CMCs through rapid identification. However, there are few reports on this aspect.

Therefore, this paper proposes a method for rapid prediction of N-CMCs properties based on IRT, XCT and a multiscale method, aiming to achieve defect detection and efficient performance assessment of N-CMC plates through IRT. First, three types of N-CMC specimens with different porosity were manufactured by adjusting the matrix deposition time. The thermal diffusion coefficient of the specimens was determined using the IRT technology and transmission flash method. Then, based on XCT technology, the pores of the composites were observed and identified, and the multiscale thermal diffusion analysis models were constructed. Furthermore, based on the multiscale models, the thermal diffusion coefficient of different porosities was predicted by simulating the boundary conditions of the thermal diffusion coefficient test. To analyze the effectiveness, the results of the IRT test and prediction were compared. The relationship between total porosity and thermal diffusion coefficient was determined. Finally, periodic boundary conditions were applied to the multiscale models to predict their elastic properties, thus recognizing the relationship between porosity and elastic properties of the composites. The accuracy of the method was verified by comparing the results of thermal and mechanical tests, as well as simulation analysis of different porosities. Finally, based on the results of model analysis, the correlation equations between thermal diffusion coefficient, porosity and elastic modulus were established. The core innovation of this work is the systematic establishment and experimental validation of a quantitative relationship model linking thermal diffusivity, porosity, and macroscopic stiffness of composite materials. Thereby, new ideas are provided for the assessment of porosity and elastic properties of the composites.

## 2. Preparation Process of Specimens

**Figure 1:**
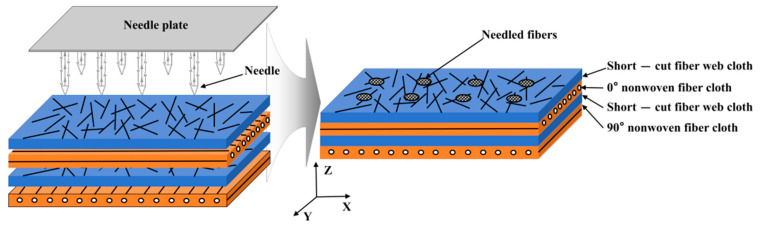
Schematic diagram of the needled fiber preforms.

The needled C/C-SiC composites used in this work were provided by the Institute of Metal Research, Chinese Academy of Sciences. Figure 1 shows the preparation process of the composite preform. The process primarily comprised the following three steps:

(1) Alternately stacking fiber composite materials such as 0° non-woven fiber cloth, short fiber web cloth and 90° non-woven fiber cloth until a specified thickness was achieved;
(2) Performing needling on the stack surface, during which the carbon fiber preform moved horizontally along the conveyor belt while the needle plate reciprocated vertically at a predetermined frequency;
(3) Rotating the composite stack horizontally by 90° and repeating the needling process to ensure uniform consistency of needle perforations along both the x- and y-axes of the material.

These three steps were repeated until the preform reached the required thickness and needling density (25 needles/cm^2^). After the needling process, excess edge material was typically trimmed from the preform to obtain specified dimensions and surface flatness.

Based on the aforementioned process, the pyrolysis carbon interface phase formed by the pyrolysis of hydrocarbon substances was deposited and attached to the surface of the carbon fiber preform at a high temperature. The precursor was then introduced into the carbon fiber preform by diffusion and convection in the deposition oven. The precursor was decomposed into the SiC matrix and deposited on the surface of the preform when heated to a certain temperature [22]. The reinforced phase in the experimental material was Toray T700 12K carbon fiber; the diameter of the carbon fiber monofilament was about 7 μm; and the thickness of the pyrolysis carbon was about 1.5 μm. Three types of specimens were obtained by adjusting the deposition time of the matrix. The porosities of specimens, determined by the Archimedes drainage method, were 10%, 20.5%, and 23%, respectively. To facilitate the subsequent experiments, the needled C/C-SiC composite plate was cut into test specimens according to the experimental standard ASTM C1275-18.

## 3. Microstructure Observation and Analysis

### 3.1. XCT Observation

X-ray tomography(Carl Zeiss, Shanghai, China) was performed using the Xradia 610 Versa 3D X-ray microscope, at the Instrumental Analysis Center of Dalian University of Technology.

According to the XCT detection principle, the smaller the pixel size, the more clearly and accurately the internal microstructure characteristics of the material can be determined. However, due to the small image area, the observed data were limited. Finally, considering the internal microstructure of the material and the specimen size, the pixel size was determined to be 5 μm. The detailed detection parameters are listed in Table 1.

**Figure 2:**
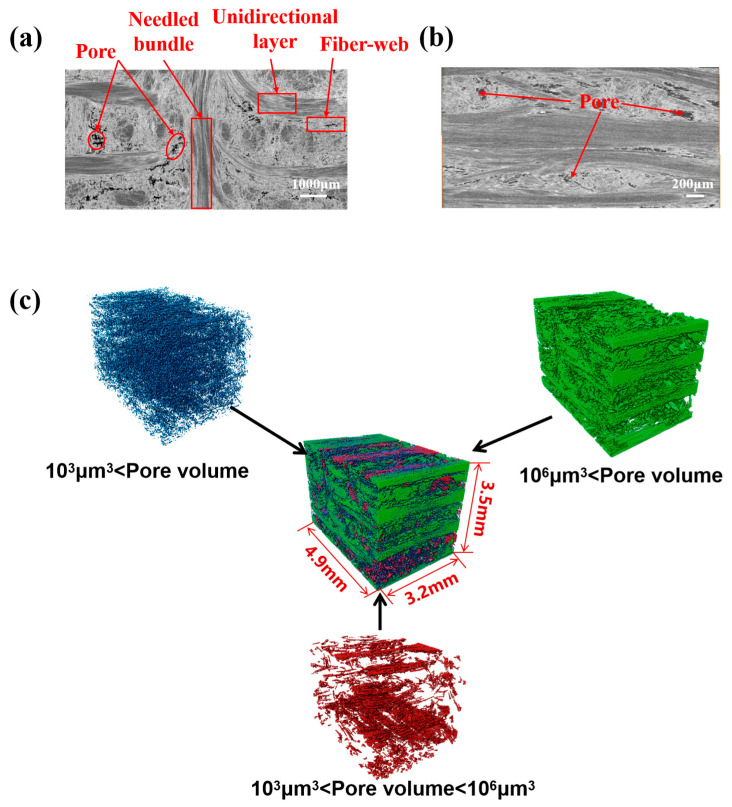
XCT images of N-CMCs (**a**) in the X-Y plane; (**b**) in the X-Z plane; (**c**) pore reconstruction images.

Considering the dispersion of the composites, the XCT was performed at two randomly selected locations. A total of 1000 2D grayscale images were obtained for each region. Figure 2 shows two typical XCT images. Among them, the difference in material composition was mainly reflected in the difference in the gray scale of the image: the higher the gray value, the higher the density, while the lower the gray value, the lower the density.

XCT images (Figure 2a,b) show the internal microstructure of N-CMCs, which was mainly composed of alternately arranged fiber-web layers and unidirectional layers, and the needle bundles passed through the direction of the composite thickness. The internal manufacturing defects of the composites were mainly reflected in the matrix pores, which had a significant scatter in size and location due to the different fiber morphologies. To further determine the characteristics of pore defects in composites, Avizo 2020 software was used to analyze their geometric characteristics, as shown in Figure 2c. In Figure 2c, blue was employed to indicate small volume pores, green represents large volume pores, and red indicates medium volume pores. The analysis revealed significant differences in pore volume and orientation between different regions.

### 3.2. IRT Observation

**Figure 3:**
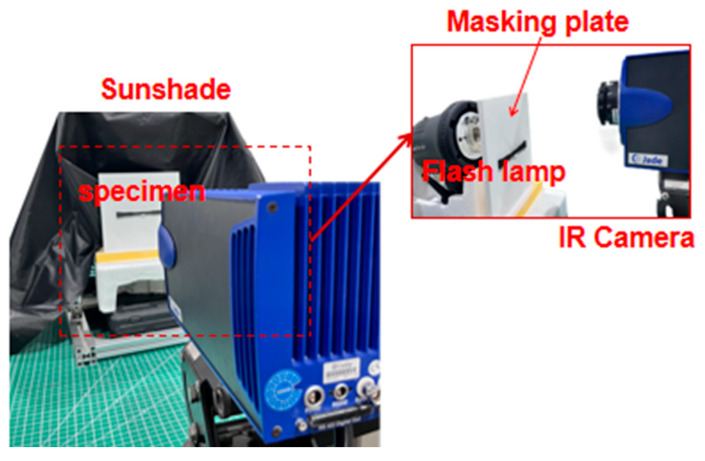
IRT test process.

The IRT detection system (Cedip, Paris, France), which mainly included an infrared thermal imaging camera, a flash excitation thermal source and a data acquisition and processing system as shown in Figure 3. The thermal imaging camera with a measuring range of 320 × 240 was the Jade infrared thermal imager. At room temperature, the noise equivalent temperature difference was only 0.025 °C. During the experiment, a cover plate and sunshade were designed to prevent the pulse excitation from affecting the detection of the thermal imager.

At the same time, in the case of pulsed thermal excitation, the influence of three-dimensional thermal diffusion was ignored, and only a one-dimensional thermal transfer model in the X direction was considered. The flash method proposed by Parker et al. [23] was used to measure the thermal diffusion coefficient of materials. For a solid material with thickness *L*, after receiving a flash energy of *Q* thermal per unit area, the surface temperature *T* of the back of the specimen was given by

(1) $$T(L,t)=\frac{Q}{\rho cL}[1+2\sum_{n=1}^{\infty }\left(-1{)}^{n}\cdot \exp(\frac{-n^{2}\pi^{2}\alpha t}{L^{2}}\right)]$$

where *L* is the thickness of the solid material; *t* is time variable; *ρ* is the density of the material; *c* is the specific thermal capacity of the material; *α* is the thermal diffusion coefficient of the composites.

Based on the above equation, two dimensionless parameters, *V* and *ω* were defined as

(2) $$\left\{\begin{array}{c} V(L,t)=\frac{T(L,t)}{T_{M}}\\ \omega =\frac{\pi^{2}\alpha t}{L^{2}} \end{array}\right.$$

where $T_{M}$ is the highest temperature of the rear surface.

The relationship between *V* and *ω* could be obtained by combining the above formulas, as shown in Equation (3).

(3) $$V=1+2\sum_{n=1}^{\infty }\left(-1{)}^{n}\cdot \exp(-n^{2}\omega \right)$$

The rapid detection method for thermal diffusivity was derived. When *V* = 0.5 and *ω* = 1.38, the formula for calculating the thermal diffusivity of the composites was obtained, as shown in Equation (4).

(4) $$\alpha =1.38\frac{L^{2}}{\pi^{2}t_{1/2}}$$

where $t_{1/2}$ represents the time corresponding to half of the highest temperature rise at the rear surface of the tested specimen.

## 4. Multiscale Model Construction and Property Prediction

### 4.1. Multiscale Model Construction

Using Avizo analysis and considering the pore characteristics of different regions, a multiscale modeling method was employed, as shown in Figure 2. At the microscopic scale, the models of fiber bundles and fiber-web layers were established, considering the shape characteristics of the pores. At the mesoscale, a mesoscale model with the fiber-web layers, the unidirectional layers and the needled bundles was constructed with reference to the classical RVE modeling method of N-CMCs [24].

#### 4.1.1. Microscale Model

At the microscopic scale, a multi-level modeling approach was proposed in this work. An equivalent matrix model was first constructed from a matrix considering the pore characteristics of different regions. Then, the fiber bundle model, including interface, fiber and equivalent matrix, was constructed. Furthermore, the fiber bundle model was employed to construct a random distribution model to characterize the fiber-web layers.

Under the framework of multi-level microscopic modeling, the equivalent matrix model was constructed using the idea of ellipsoid fitting according to the analysis results. The equivalent diameter, the aspect ratio and the pore direction were selected as the characterization variables of the pores.

The detailed construction processes are as follows. The pore characterization parameters of different regions were determined by Avizo. Then, the probability density function of pore characterization variables was obtained by fitting the function. Finally, the Monte Carlo accept–reject sampling method was selected to describe the pore defect distribution consistent with the fitting function.

**Figure 4:**
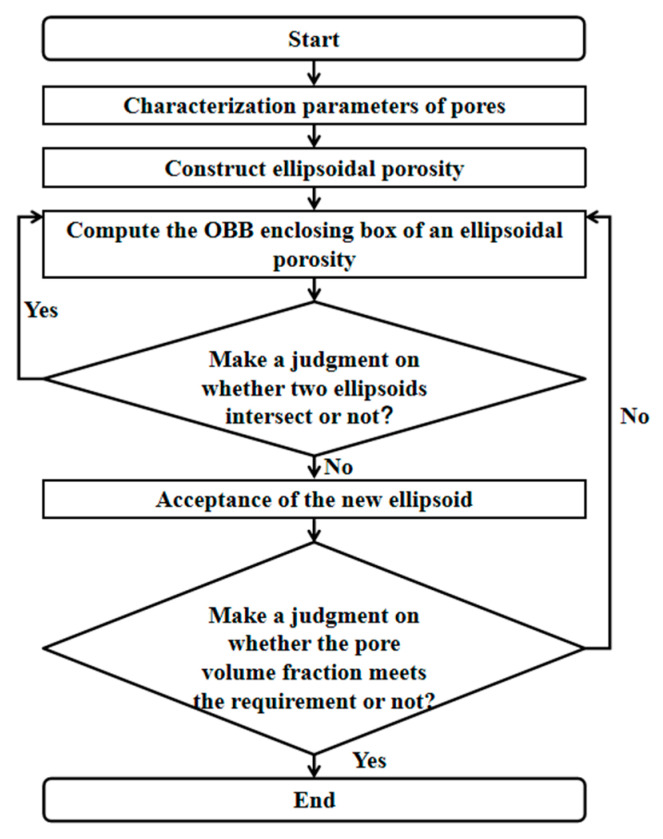
Ellipsoidal pore modeling flow chart.

**Figure 5:**
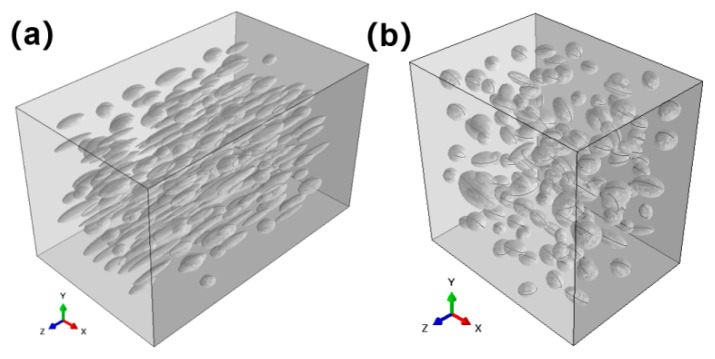
Equivalent matrix models considering pores: (**a**) the unidirectional layers; (**b**) the fiber-web layers.

After the basic structural parameters of ellipsoidal pores were determined, the modeling process shown in Figure 4 was used to construct the finite element model in Abaqus 2025 based on Python 3.14.0, as shown in Figure 5.

**Figure 6:**
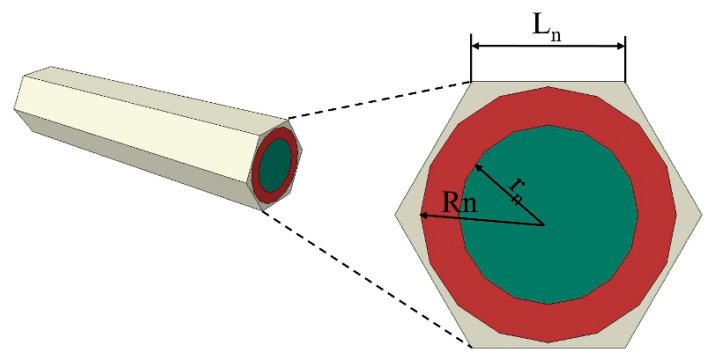
The fiber bundle model, where Rₙ is the radius of the interface plus the fiber; L_n_ is the side length of the hexagonal RVE, and rₙ is the radius of the fiber.

Then, according to the structural characteristics of the fiber bundles in different regions, the fiber bundle model and the fiber-web model were constructed, respectively. Among them, the fiber bundle model was suitable for the unidirectional layers and the needle bundles. In this work, the hexagonal RVE was employed to simulate the fiber bundle layout, where green represents the fibers, red represents the interfaces, and the outermost part is the equivalent matrix, as shown in Figure 6 [25,26].

**Figure 7:**
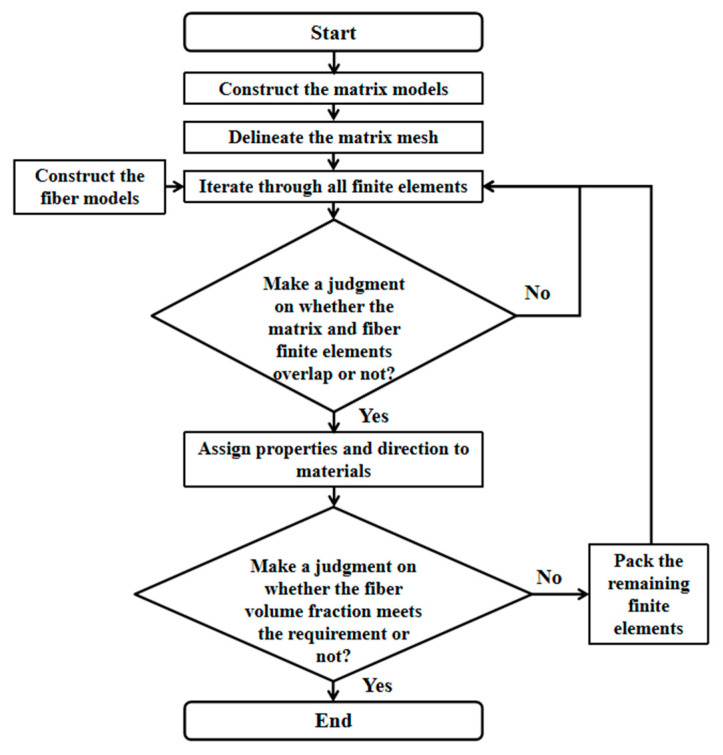
Flow chart of fiber-web layer modeling.

**Figure 8:**
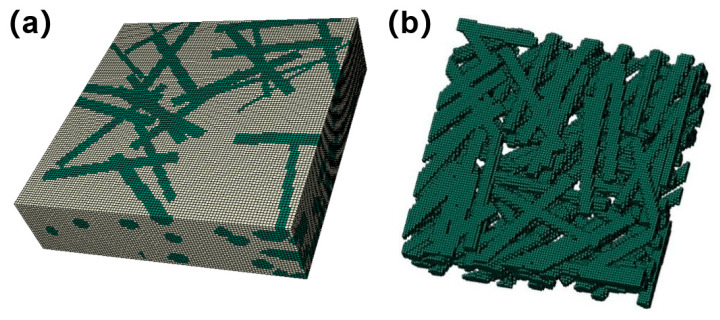
Model of the fiber-web layers by Voxel method: (**a**) yellow with matrix; (**b**) green random short fibers.

In order to realize the modeling of a high fiber volume fraction for the fiber-web layers, taking the actual microstructure information into account, the random sequence adsorption (RSA) method was combined with the Voxel meshing technology to establish the analysis model of a short fiber mesh. The specific implementation process and model are shown in Figure 7 and Figure 8. Among them, the properties of the fiber-web layers were derived from the fiber bundle prediction results described above.

#### 4.1.2. Mesoscale Model

**Figure 9:**
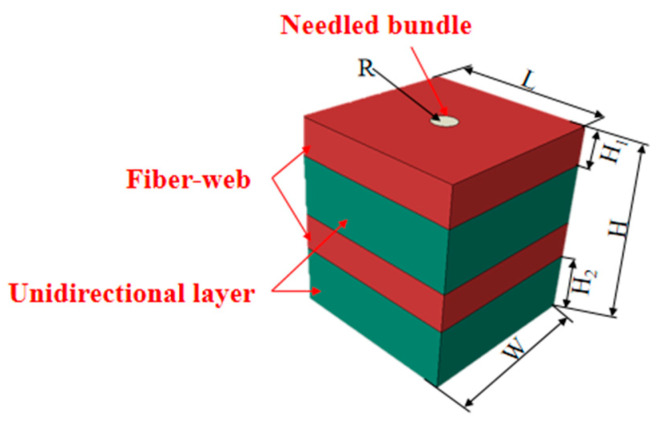
Mesoscale RVE model, where the fiber-web layers are shown in red, the unidirectional layers in green, and the needle bundles in yellow. *R* denotes the radius of the needle bundles; *L* is the length of the mesoscale RVE model; *W* is the width; *H* is the total thickness; *H*_1_ and *H*_2_ represent the thicknesses of the fiber-web and unidirectional layers, respectively.

Referring to the classical mechanical model of N-CMCs, the mesoscale model of N-CMCs was established, as shown in Figure 9. The model also consisted of typical structures of the fiber-web layers, the unidirectional layers and the needle bundles. Among them, the fiber-web layers, the 0° unidirectional layers and the 90° unidirectional layers were arranged alternately in the direction vertical to the composite thickness, and the cylindrical needle bundles passed through the direction of the composite thickness.

### 4.2. Property Prediction

**Figure 10:**
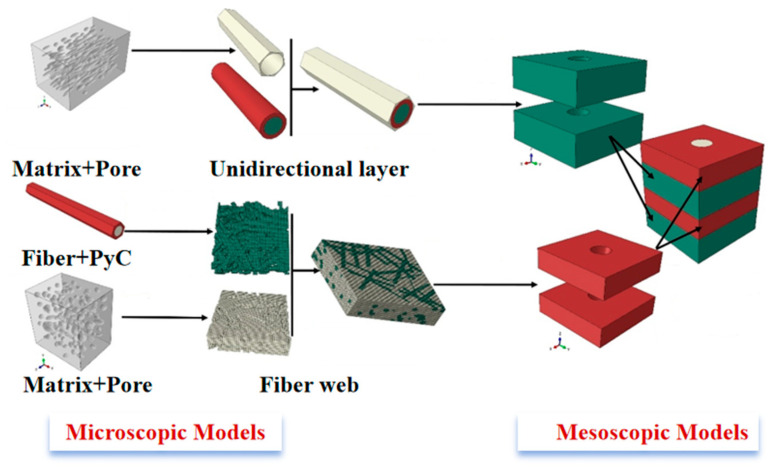
Multiscale prediction method on the N-CMC properties.

The Micro-Mechanics plugin of Abaqus was used to calculate the equivalent elastic properties of models containing various structures at the microscopic and mesoscopic scales, respectively. The equivalent parameters obtained after homogenization at the microscale model were input into the mesoscale and macroscale models. The elastic properties of N-CMCs could be determined considering various structural characteristics, as shown in Figure 10. At the same time, the thermal diffusion coefficient of composites cannot be obtained directly using the Micro-Mechanics plugin, nor is it suitable for calculation using the theoretical method. Therefore, a thermal flow excitation with an intensity of 4 × 106 W/m^2^ and a duration of 0.6 ms was applied to the bottom of the model to simulate the detection boundary of the thermal diffusion coefficient using the flash method. Finally, the thermal diffusion coefficient of the model was obtained by extracting the temperature rise curve of the top element.

## 5. Results and Discussion

### 5.1. Defect Analysis and Parameter Characterization

**Figure 11:**
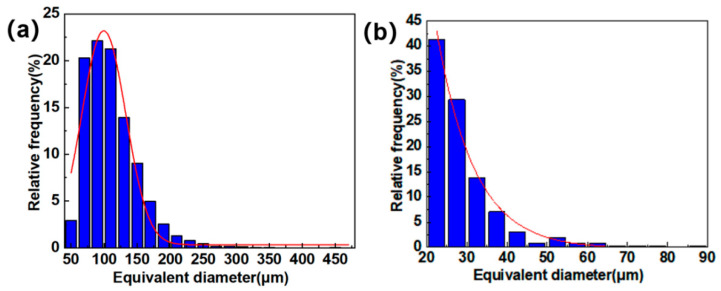
Pore equivalent diameter distribution: (**a**) the fiber-web layers; (**b**) the unidirectional layers.

In this work, the pores in different regions were extracted and characterized separately, as shown in Figure 2c. Figure 11a,b show that the equivalent diameter of large pores in the fiber-web layers was concentrated around 100 μm, and the pore defects with an equivalent diameter of more than 200 μm accounted for about 3% in total. The Gaussian curve fitting effect was better for the distribution, as shown by the red curve in Figure 11. In addition, the equivalent diameter distribution map of the unidirectional layers was better fitted by the exponential function. The equivalent diameter, whose size was obviously smaller than that of the fiber-web layers, was relatively concentrated and mainly distributed between 20 μm and 40 μm.

**Figure 12:**
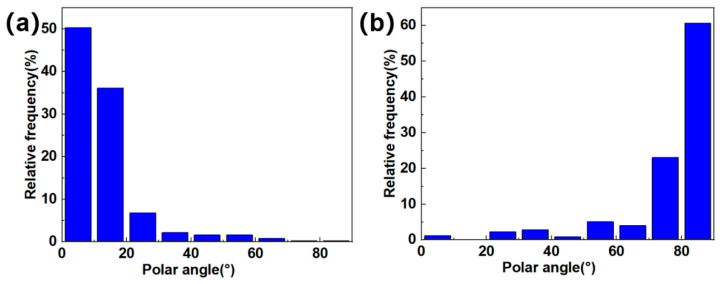
Distribution of pore polar angles in 0° unidirectional layers (**a**) and 90° unidirectional layers (**b**).

There was no significant peak for the angular distribution of the pores in the fiber-web layers. Therefore, the uniform distribution was used when modeling the pore direction in the fiber-web layers. However, the polar angle of the pores in the unidirectional layers had an obvious directional dependence. Figure 12 shows the distribution of pore polar angles in 0° and 90° unidirectional layers. It can be seen that the pore angle distribution was relatively concentrated, and the pore direction distribution was concentrated in the fiber direction.

**Figure 13:**
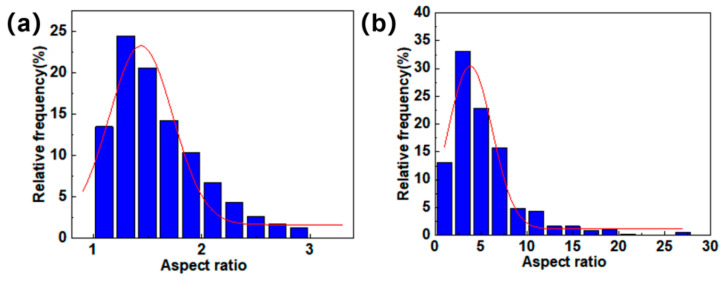
The aspect ratio of pores: (**a**) fiber-web layers; (**b**) unidirectional layers, where the red curves represent the Gaussian fitting curve.

Figure 13a shows that the aspect ratio of the pores in the fiber-web layers was predominantly below 2, and the pores had a nearly spherical shape. According to Figure 13b, the aspect ratio of the pores in the unidirectional layers was mainly distributed between 3 and 7, which was significantly larger than that of the fiber-web layers and had a better concentration ratio.

### 5.2. Thermal Diffusivity Detection

**Figure 14:**
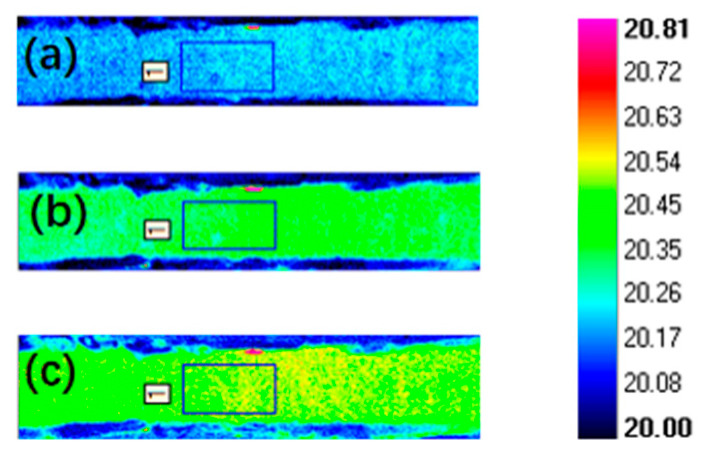
Surface temperature distribution of the specimen by the IRT test (**a**) at the initial stage of applied pulse excitation; (**b**) during temperature rise; (**c**) when the temperature reached the highest point.

**Figure 15:**
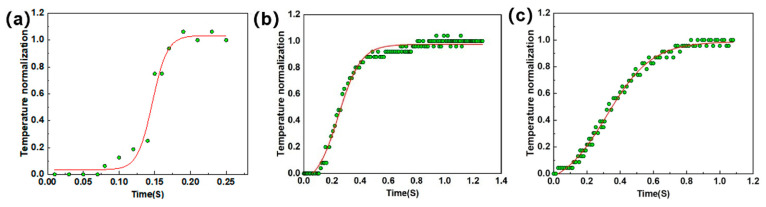
Temperature rise fitting curves for specimens with different porosity: (**a**) 10%; (**b**) 20.5%; (**c**) 23%, where green dots are collection points and red lines are fitting curves.

The variation in the surface temperature field of the specimens was captured by the thermal imager during the experiment, as shown in Figure 14. After obtaining the image data of the temperature rise on the back of the specimen, the Altair software developed by the Cedip company in France was used for data analysis. Altair software was used to automatically calculate the average temperature of the region at a certain frame rate, and the thermal diffusion coefficient of the material was calculated by combining the half-height time method, as shown in Figure 15.

The calculations of thermal diffusion coefficient detection of the specimens with different porosities are listed in Table 2.

### 5.3. Thermal Diffusion Coefficient Simulation Analysis

**Figure 16:**
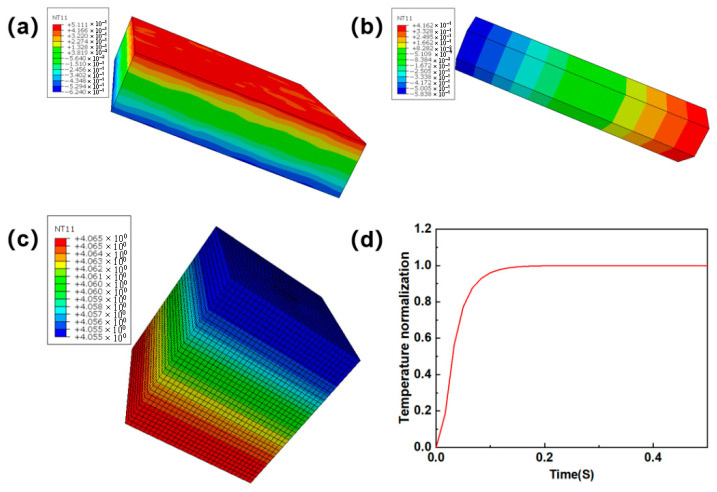
Finite element analysis results of the RVE model with 10% porosity: (**a**) thermal diffusion in the fiber-web layers; (**b**) thermal diffusion in unidirectional layers; (**c**) mesoscale model thermal diffusion; (**d**) fitting curve of excitation temperature rise.

Based on Table 3, combined with the multiscale analysis method, the regional analysis contour and temperature rise curve were gradually calculated and determined, as shown in Figure 16.

According to the above temperature-rise fitting curve, the time corresponding to half of the curve was determined and then substituted into Equation (4) to solve the thermal diffusion coefficient of the model at room temperature. The analysis results of the thermal diffusion coefficient at different porosities are listed in Table 4. The findings indicated that the prediction error for specimens with 10% porosity was 8.07%, while that for specimens with 23% porosity reached 9.06%, demonstrating favorable predictive accuracy. The deviation was mainly attributed to the combined effect of non-uniform local pore distribution and variations in needling density, which jointly induced structural heterogeneity at the microscale and changed the effective heat transfer paths in a manner that was not fully represented by the idealized model. Empirical studies confirmed that this multiscale methodology effectively achieved precise prediction of thermal diffusivity.

### 5.4. Elastic Modulus Prediction

In the process of finite element calculation, the main structural property parameters and experimental values are listed in Table 5. The predicted and experimental results of the elastic modulus with 10% porosity specimens are shown in the table. It can be seen that the error of the multiscale prediction method was 3.1%. This minor deviation was mainly due to the non-uniform distribution of local pores and variations in needling density, as previously identified, which caused local fluctuations in stress concentration and load transfer. It showed that the multiscale approach has good accuracy in predicting the elastic modulus of the composites.

### 5.5. Correlation Analysis and Application

**Figure 17:**
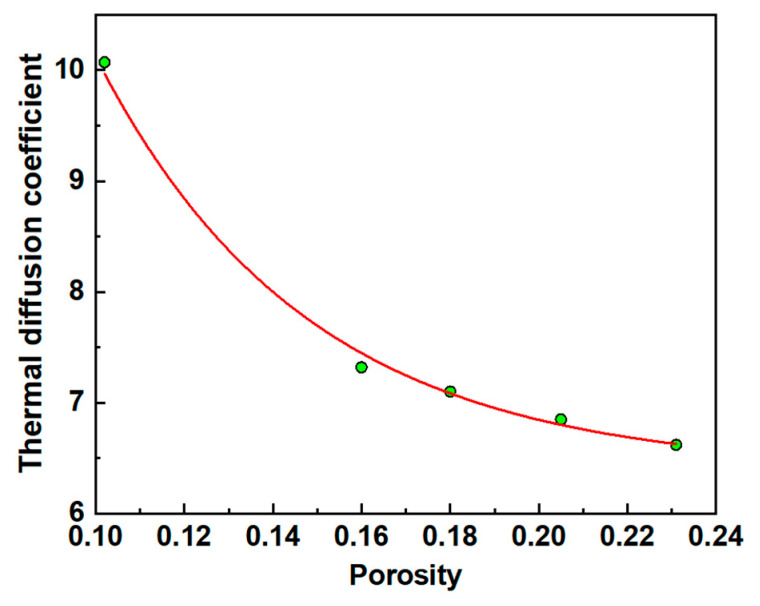
The variation tendency of porosity and thermal diffusion coefficient, where green dots are predicted points and red is fitting curve.

The porosity and thermal diffusion coefficient obtained by finite element analysis were fitted, and the iterative algorithm of orthogonal distance regression was used to determine the quantitative correlation between porosity and thermal diffusion coefficient. The fitted variation curve of thermal diffusion coefficient with porosity is shown in Figure 17.

It was found that the thermal diffusion coefficient decreases with increasing porosity, showing a decreasing exponential trend. The fitting function of its change rule was given by

(5) $$y=6.39+30.52e^{-\frac{x}{0.05}}$$

where y is the thermal diffusion coefficient; *x* represents the porosity of the composite materials; e is the Euler’s number.

By substituting the thermal diffusion coefficient obtained from the IRT test into the fitted curve of porosity versus thermal diffusion coefficient in Equation (5), the porosity corresponding to the different thermal diffusion coefficients could be obtained inversely. Taking the specimen with a porosity of 20.5% as an example, Table 4 shows that the thermal diffusion coefficient 6.82 mm^2^/s was used in Equation (5) for this porosity. The porosity in the numerical model was about 21.31% with a comparison error of 3.95%.

The porosity was introduced into the multiscale model. Then, the elastic properties of the material were calculated using the finite element method, and the test specimens with the same porosity were verified by experiments. The corresponding results are listed in Table 6.

By comparing the results, it can be seen that the difference between the predicted value and the experimental value is about 2.8%, which shows excellent consistency. The results show that the quantitative relationship model between thermal diffusivity, porosity and macroscopic stiffness constructed by this method can achieve rapid defect assessment and performance prediction of composite material plates in a non-destructive state, and has a wide range of application prospects.

## 6. Conclusions

(1) The pore shapes and sizes of N-CMCs exhibited strong multiscale characteristics and dispersion. However, due to the various fiber morphologies in different regions, the pore size, direction and aspect ratio corresponded to a certain function distribution.
(2) The multiscale analysis method proposed in this paper, which fully considered the geometric and distributional characteristics of the pores, provided better accuracy in predicting the thermal diffusion coefficients and the elastic modulus. It should be noted that the model developed in this study was primarily validated against laboratory-scale standard specimens. When applied to large components with complex geometries, challenges such as increased blind spots in infrared detection and potential distortions in thermal conduction simulations may arise, which could have a certain impact on the prediction accuracy.
(3) Based on the prediction results, it can be seen that there is a mapping relationship between porosity, thermal diffusion coefficient and elastic modulus. By using IRT to test the thermal diffusion coefficient in combination with the multiscale analysis method, the rapid identification and accurate prediction of the porosity and elastic modulus of the composites can be achieved.

## Figures and Tables

**Table 1:** Test parameters of X-ray tomography.

Voltage (kV)	Electricity (μA)	Angle (°)	Exposure Time (ms)	Pixel Size (μm)
50.0	90	5.32	1000	5.0

**Table 2:** Experimental results of the thermal diffusion coefficient of specimens with different porosities.

Porosity	Thickness (mm)	t_1/2_ (s)	Thermal Diffusion Coefficient (mm^2^/s)
10%	3.60	0.175	10.36
20.5%	3.63	0.27	6.82
23%	3.61	0.30	6.07

**Table 3:** Material parameters of different components of CMCs [26].

Properties	Longitudinal Thermal Conductivity W/(m·K)	Transverse Thermal Conductivity W/(m·K)	Specific Thermal (J/kg·K)	Density kg/m^3^
Carbon fiber	9.4	3.6	752	1800
Matrix	23.4		700	3200
Pyrolysis carbon	25		717	1800
Pore	0.023		1005	1.29

**Table 4:** Results of finite element prediction of thermal diffusion coefficient at different porosities.

Porosity	Thermal Diffusion Coefficient (mm^2^/s)	Experimental Value (mm^2^/s)	Error
10%	10.07	10.36	2.85%
23%	6.62	6.07	9.06%

**Table 5:** Prediction results of elastic properties of CMCs.

Model	E1/(GPa)	E2/(GPa)	E3/(GPa)
Fiber-web layers	56.76	52.52	58.90
Unidirectional layers	111.56	16.09	14.19
RVE model	55.96	52.92	26.09
Experiment value	57.76		

**Table 6:** Prediction and experimental results of elastic properties of composites with 20.5% porosity.

Porosity	E1 (GPa)	E2 (GPa)
Fiber-web layers	40.77	31.01
Unidirectional layers	81.24	8.38
RVE model	39.56	35.55
Experiment value	40.69	

## Data Availability

The original contributions presented in the study are included in the article, further inquiries can be directed to the corresponding author.
